# Tailored Teaching for Specialized (Para-)medical Students - Experience From Incorporating a Relevant Genetic Disease Throughout a Course of Molecular Cell Biology

**DOI:** 10.3389/fpubh.2020.00224

**Published:** 2020-07-09

**Authors:** Ton Schoenmaker, Dongmei Deng, Teun J. de Vries

**Affiliations:** ^1^Department of Peridontology, Academic Centre for Dentistry Amsterdam (ACTA, University of Amsterdam and Vrije Universiteit), Amsterdam, Netherlands; ^2^Department of Preventive Dentistry, Academic Centre for Dentistry Amsterdam (ACTA, University of Amsterdam and Vrije Universiteit), Amsterdam, Netherlands

**Keywords:** course innovation, fibrodysplasia ossificans progressiva, molecular cell biology, osteoclast, periodontal ligament fibroblast, heterotopic ossification, medical education

## Abstract

Worldwide, a mandatory course in Molecular Cell Biology is often part of the (para-) medical curricula. Student audiences are regularly not receptive to such relatively theoretical courses and teachers often struggle to convey the necessary information. Here, positive experience is shared on rigorously embedding a genetic disease that severely affects the movement apparatus, fibrodysplasia ossificans progressiva (FOP), in all aspects of a course for an international group of Research Master Human Movement Sciences students. Various molecular cell biological aspects of FOP were systematically implemented in the course, covering genetics, the biochemical consequences of the mutation, signaling pathways that affect bone formation and lectures on how to clone the mutation or cure the mutation. Students were invited to critically think about how to use the theories learned in the course to analyze a research paper. During the practical part of the course, students assisted in novel, cutting edge research on FOP patient derived or control cells. Research findings were reported in a research paper format. By building a Molecular Cell Biology course around an appealing disease, we managed to increase the general motivation of the students for the course as reflected in two specific questions of the course evaluations (*p* < 0.05). It convincingly taught the relevance of a course of Molecular Cell Biology to students with a primary background in biomechanics and physiotherapy for their paramedical professional life. This approach of embedding an audience-tailored human disease with a known genetic cause into a course can be implemented to many medical curriculum related courses and will increase students' perception of the relevance of a course.

## Introduction, Objectives

It is probably a widespread and recognizable experience for many teachers of courses in the medical curriculum, that when finishing the course we like most of our repertoire, receive wonderful acknowledgments from our students, but in those acknowledgments we get a considerable proportion of students who ask the question we do not wish to hear: “Thanks for the nice course, but what is in it for us? I do not see how it fits into the program.” Despite the wonderful course evaluations received, such questions keep on nagging for some time. We have organized a molecular cell biology course for Human Movement Sciences students. Although most aspects of the course evaluation scored satisfactorily from the start and gradually further improved, this question, “What's in it for us?” persisted until by chance we got involved in research material of a disease that appealed to the students as it severely affects the movement of these patients. This allowed us to integrate this disease, fibrodysplasia ossificans progressiva (FOP), fully in virtually all aspects of the Molecular Cell Biology course. By this rigorous integration, the course lost its level of abstraction and came closer to all students. It uplifted the course further, resulting in students who kept their interest and attention throughout the course and in teachers that could tell a logical follow-up story. Here, we would like to share this positive experience, which we believe can be extrapolated to other courses of the paramedical curriculum.

## Methodology

### The Students, the Course, the Position in the Program, the Challenges

Around 10 years ago, a 2-years Research Master's program Human Movement Sciences has seen the light at the Faculty of Human Movement Sciences at the Vrije Universiteit (VU) Amsterdam in the Netherlands. For this international English taught program, 15–25 top students per year are selected based on grade average of their Bachelor's degree and a motivational interview. From its founding, Molecular Cell Biology was a part of the curriculum for three European Credits (ECTS), which is roughly 1.5 American Credits, or 2 weeks full time-basis equivalent, where 1 ECTS = 28 h. For most students, Molecular Cell Biology is the first encounter of a course in that category and therefore non-central to what they had learned before. Human Movement Scientists primarily have a background in biophysics, biomechanics, neuroscience, and physiotherapy. The course has always contained a theoretical part and a practical part of one and a half days, during which students actively participated in laboratory work, isolating RNA and performing quantitative real-time PCR (qPCR) of genes of interest. A written report that has the format of a research article was the requested output format for the practical, which made up one third of the grade. A written exam on the theory made up for the remaining two thirds of the final mark. The challenges have always been two-fold: to bridge the theoretical part with the practical, which until a few years ago was a separate entity, and to convince students of the broader context of Molecular Cell Biology, such as how to apply it to a disease-related research question. The course has long been considered an unusual requirement of the curriculum of the Research Master of Human Movement Sciences, as compared to the other courses.

### The Disease FOP

As a coincidence, some 5 years ago, we were approached for culturing cells from extracted teeth, from a patient with fibrodysplasia ossificans progressiva (FOP). The VU medical center is the FOP center of expertise in the Netherlands [link: https://www.vumc.com/departments/fop-amsterdam-info-research-and-trials/fop-patient-care-vumc.htm:]. Teeth were extracted from patients with a closing jaw joint for the purpose of creating more space, such as in patients with a bony bridge that locks the jaw joint (1). All material was surgical waste material and permission to culture cells for research purposes was obtained through informed consent and further institutional ethical approval. The disease manifests itself as a disease with abnormal progressive bone formation of the connective tissue, hence the name. Especially connective tissue of tendons, ligaments, and muscles turn into bone. Gradually, the ability to move declines. Patients often end up in a wheelchair, see for instance this informative clip on YouTube, presented by the FOP specialist Dr. Frederick Kaplan [link: https://www.youtube.com/watch?v=GksggHYAA7M]. The causative mutation was discovered in 2006 (2). This one in two million occurring dominant mutation is located in ALK-2 or ACVR-1, a subunit of a bone morphogenesis protein receptor. To sum up, by this mutation, the off-switch of bone formation is defective. It was only in 2015 that the preferred ligand Activin A for this mutant ACVR-1 was discovered (3, 4). By this ligand-receptor interaction, pSmad1/5/8 signaling is elevated specifically by the mutated ACVR-1. Also an inhibitory molecule, FKBP12 does not interact properly with the mutated ACVR-1, allowing leaky signaling. Together this results in ectopic bone formation in FOP patients, disabling their possibility to move properly. Our involvement in this research came as a blessing in disguise, since now we could fully integrate a disease that severely impairs the movement apparatus into our course for human movement scientists, highly relevant for the future work field of students who will deal with patients with movement impairment. When supervised properly, students could even participate using the patient-derived and control cells in the practical. It further synergized expertise at Amsterdam Medical Center of the Vrije Univeristeit (VUmc) and at Academic Centre for Dentistry Amsterdam (ACTA), where expertise in tooth-associated osteoclast formation (5) and osteogenesis exists (6). Since taking a biopsy could lead to a flare-up inducing heterotopic bone formation, this is an absolutely forbidden procedure in FOP clinical research. Therefore, traditionally, relevant patients derived cell models has always been cumbersome. Cells obtained from teeth have been explored to some extent (7, 8). Extracted teeth are surrounded by an ultrathin layer of periodontal ligament fibroblasts, which can be readily cultured and propagated. Since these are cells from a true ligament, anchoring teeth into the bone, they are potentially a valuable cell model to study FOP associated bone remodeling processes.

## Results

### A Disease Integrated Course of Molecular Cell Biology

The course became a disease-integrated course of Molecular Cell Biology and students benefited from this approach by connecting aspects of Molecular Cell Biology to a disease relevant for them. The course also provided the opportunity to contribute to cutting edge research. When using chapters of regularly used text books such as *Essential cell biology* (9) the incorporation of the Molecular Cell Biology of the disease FOP was relatively easy (See Table 1).

**Table 1:** Example of course schedule of a Molecular Cell Biology course and how a disease, here FOP, can be embedded throughout.

**Activity**	**Subject**	**Integration of the disease FOP in the course**
Class 1	Introduction of the course DNA- RNA- Protein central dogma of Molecular Biology	Introduction of disease, with movie from internet, take time to discuss all patient related aspects of disease. How to explain inheritance of a dominant gene when parents are not affected?
Class 2	Structure of genes RNA transcription and modulation	Structure of ACVR-1 gene Structure of ACVR-1 mRNA transcript and protein
Class 3	Molecular techniques I Sequencing Cloning	How does a mutation in ACVR-1 gene affect the cell function? Strategies for cloning and bio-assays for normal and mutated ACVR-1
Class 4	Molecular techniques II QPCR Microarray/RNAseq	Gene expression comparison (one or multiple genes) between healthy cells and cells with FOP mutation. Display own results of RNAseq control vs. FOP
Class 5	Signaling Flow of information from outside cell toward effects inside General aspects of signaling	Signaling with normal and mutated ACVR-1 and its negative regulator FKBP12 and downsteam molecules pSmad1/5/8, role of Activin A
Seminar	Consolidation of knowledge, both Molecular cell biology and FOP: Reading a research paper together.	A research paper on FOP such as when osteoclasts were central issue (10); or on Activin A (3).
Class 6	Bridge to practical	Explain the experiment, qPCR, and what is expected during practical on FOP patients derived cells.
Practical	RNA isolation, cDNA synthesis and qPCR of genes expressed by fibroblasts or in co-cultures of fibroblast-and osteoclast precursors Students learn laboratory techniques and how to calculate expression data in one and a half day	Control vs. FOP, such as published in (11–13). Typical genes to be assessed: ACVR-1, FKBP12, bone formation proteins RUNX-2; Osteopontin, Osteocalcin, Alkaline phosphatase, and osteoclast genes TRACP, DC-STAMP.
Exam	On theory Class 1–5 and research paper Class 6	Both questions on general molecular cell biology and related to FOP.

Teachers experienced a tremendous difference when teaching this course compared to the years before a disease was embedded. Remarkably, when critically analyzing the changes, ~80% of the course remained the same. It was easier for one teacher to continue where the other teacher had left, primarily because the disease was the main theme of the course. Students incorporated Molecular Cell Biology at a level that was relevant for them, since the overarching disease was in the limelight, as an example of a disease effecting the movement apparatus.

The practical, which was without exaggeration announced in phrases like “you can contribute to unique work that has not been performed yet and outcomes are relevant for our understanding of the disease” nicely connected to the theory. Students following the Research Master of Human Movement Sciences are supposed to write a research paper in their 2nd year, based on their individual 1-year long research project. The course helped them to prepare for this, but now in a group setting, exploiting the individual talents of students such as writing, structuring data and final editing of a text. In the last couple of years, we assessed bone formation markers in these unique cells from FOP patients and also osteoclast markers in co-cultures of these cells and peripheral blood cells. Results contributed by the students have become part of two publications (11, 12) and it is foreseeable that they will do so in some future publications. Through the years, we have addressed differences in gene expression between control and FOP derived cell periodontal ligament fibroblasts, or osteoclasts. Since Activin A seems the disease specific activator, recent years have investigated its role in osteoclast formation and osteogenesis.

Pedagogically, our course set-up has similarities with novel teaching methods such as (hybrid forms of) Problem Based Learning (PBL), which seems as a more attractive way to teach medical students complex diseases from many perspectives (14).

### Motivation and Relevance Improved

The Vrije Universiteit Amsterdam has a thorough tradition of evaluating each course. Students are asked to fill-out the course evaluation a couple of days after finalizing the course. Over the years, this questionnaire has varied to some extent, but it has always contained ~20 questions on course content, course organization, student commitment, quality of the teachers, and on the exam. We specifically analyzed the effect of implementing a disease on two items of the yearly course evaluations by comparing two items before (3 years) and after (4 years) embedding a disease. These items were: (1) It was an interesting course and (2) The relevance of the course to the program was clear to me. Both aspects scored significantly higher after incorporation (Table 2). Besides the generic questions, the opportunity is provided to ask additional questions which seemed relevant for the changes that were implemented. One question was: “We deliberately incorporated a disease, FOP, that affects the movement apparatus throughout the course. Did it help to make Molecular Cell Biology more lively?” We asked this question in the year in which we first implemented the integration of FOP throughout the course and his question received a 4.68 out of 5 +/– 0.58 (S.D.) from the 19 responders.

**Table 2:** Effect of incorporation (before and after) of a disease in experiencing relevance of the course, 5-point scale.

**Question**	**Before (*n* = 3 years) Response: 13 ± 6 (S.D.)**	**After (*n* = 4 years) Response: 13 ± 5 (S.D.)**	***p*-value (*t*-test)**
It was an interesting course	3.62 +/– 0.42 (S.D.)	4.46 +/– 1.29 (S.D.)	0.0366
The relevance of the course to the program was clear to me	3.20 +/– 0.45 (S.D.)	4.11 +/– 0.35 (S.D.)	0.0266

## Discussion and Conclusions

### Translatability to Other Courses

When considering the enormous impact of incorporating this disease into how this course was experienced by both students and teachers, we would like to put our experience into a much broader perspective. Teacher-researchers should be encouraged to make their courses tailor-made to the specific (para-)medical audience they face. For instance, some of us (TJdV) have experience in teaching oral hygienist students histology of the oral tissues. For quite a few, this is a challenging and possibly even boring subject from beginning to the end, since such an audience is much more practically oriented. But, when designing a seminar where genetic defects that affect protein *function* have a disastrous outcome for the patient, histology can be approached at a different, integrated level, since the proteins do not function properly and hence the tissue structure is disrupted. This way, knowledge on histology is conveyed as extremely relevant for the working paramedical professionals. Likewise, then in a more extensive course with more techniques, we have successfully engaged students in ongoing research (15). Compared to ~30 years ago, when the genetic causes of diseases were still in their infancy, we as scientist-teachers now have the opportunity to make courses much more *audience-tailored* for specialized (para-)medical specialists. This provides tantalizing opportunities to explain anatomy, histology, cell biology, biochemistry, and molecular biology making use of the genetic and biochemical knowledge of relevant diseases. When incorporating relevant diseases, such courses increase in relevance.

## Data Availability Statement

The datasets generated for this study are available on request to the corresponding author.

## Author Contributions

TV initiated writing. TS and DD contributed their parts to the present manuscript. All authors agree on the current version of the manuscript.

## Conflict of Interest

The authors declare that the research was conducted in the absence of any commercial or financial relationships that could be construed as a potential conflict of interest.
